# Challenges of cancer imaging in Africa

**DOI:** 10.1186/1470-7330-14-S1-O2

**Published:** 2014-10-09

**Authors:** Zarina I Lockhat, Leon J van Rensburg, Hanlie du Toit

**Affiliations:** 1 Department of Radiology, University of Pretoria, South Africa; 2 Department of Radiology and Diagnostics, University of Western Cape, South Africa; 3 Drs SCP Inc, Diagnostic Radiologists, Cape Town, South Africa; 4 Department of Clinical and Radiation Oncology, VI Oncology, Cape Town, South Africa

## 

Africa as a continent is subdivided into different regions, Northern, Eastern, Western, Middle and Southern Africa. Sub-Saharan Africa refers to the combined Eastern, Middle, Southern, and Western regions. These subdivisions are important as the disease and cancer spectrum found in specific regions is quite different. Similar to the differences found between Africa and the developed world, cancer incidence and mortality patterns vary remarkably across regions within Africa because of the substantial differences in economic development, and social, cultural and other environmental factors, including major known risk factors [1].The occurrence of cancer in Africa varies remarkably by type of major cancer, stage at diagnosis, survival, incidence and mortality rates. This is largely due to differences in exposure to major risk factors, detection practices (availability of diagnostic and screening services), awareness of early signs and symptoms, and availability of treatment [1].

The cancer burden of each region will be discussed and some of the factors related to cancer imaging and the challenges faced will be highlighted.

Recent statistics and data for some parts of Africa are lacking, relatively representative in other parts, however more available for sub-Saharan Africa, which will probably be the greater focus of the discussion.

There are many significant global organisations that are constantly working to alleviate the cancer burden, and conduct research. There is significant vendor interest and investment in imaging equipment, and therapeutic modalities in scattered parts of Africa, although more focused in certain parts of Africa.

There is major local and international focus in establishing cancer centres in Africa, yet the predicted increase in the cancer burden, is phenomenal.

Collaborative and novel solutions are being explored through initiatives that involve international, regional and local organisations. These programmes include the procurement, installation, and maintenance of diagnostic and therapeutic imaging equipment.

There is much to be done, in the realm of screening, diagnosis, training, teaching, treatment and research, and this has to be an on-going, focused, enthusiastic effort by all countries in sub-Saharan Africa, the entire continent of Africa and key global cancer bodies. Much has been done in the last few years however much more still has to be done. As Victor Kgomoeswana highlights in his book “Africa is open for business”, Africa needs investment.
